# Regional rotation of the left ventricle in healthy and cardiomyopathic subjects measured with radial myocardial tagging

**DOI:** 10.1186/1532-429X-16-S1-P24

**Published:** 2014-01-16

**Authors:** Razieh Kaveh, Abbas N Moghaddam, Sarah N Khan, J Finn Paul

**Affiliations:** 1 Biomedical Engineering, Tehran Polytechnic, Tehran, Iran, Islamic Republic of; 2 Radiological Science, David Geffen School of Medicine at UCLA, Los Angeles, California, USA

## Background

Left ventricular rotational deformation, which arises from contraction of myofibers arranged in a helical structure, plays a crucial role in cardiac mechanics. Myocardial dysfunction in cardiomyopathies is usually associated with altered diastolic rotation [1-3]. However, regional myocardial abnormalities in various cardiomyopathies may result in regional alterations of both systolic and diastolic rotational motion. The regional variation of LV rotation in healthy subjects has been previously studied [4-7]. We hypothesize that LV regional rotation abnormalities may be a sensitive marker in heart diseases where myocardial structure is disordered. In this regard, we investigate the regional rotation pattern of the mid LV wall in both healthy subjects and patients with cardiomyopathies. In this study, LV regional rotation is assessed through dense radial tagging [8], which facilitates analysis of the rotational motion and provides detailed regional measurement by increasing the achievable circumferential resolution.

## Methods

LV tagging in a dense radial pattern was performed at the mid LV short axis level in twelve healthy subjects and nine cardiomyopathic patients at 1.5T or 3.0T. Number of radial taglines was set to 22 per circle. Corresponding short axis cine images were available in all subjects. The mid LV short axis was divided into 6 circumferential segments according to the AHA 17-segment model. To compute rotation of each specific segment, the tag points located on the segment were automatically detected and traced through successive frames. Finally, the rotation of each LV sector was estimated using the spatial coordinate of the tag points plus that of the LV center of mass.

## Results

**Figure 1:**
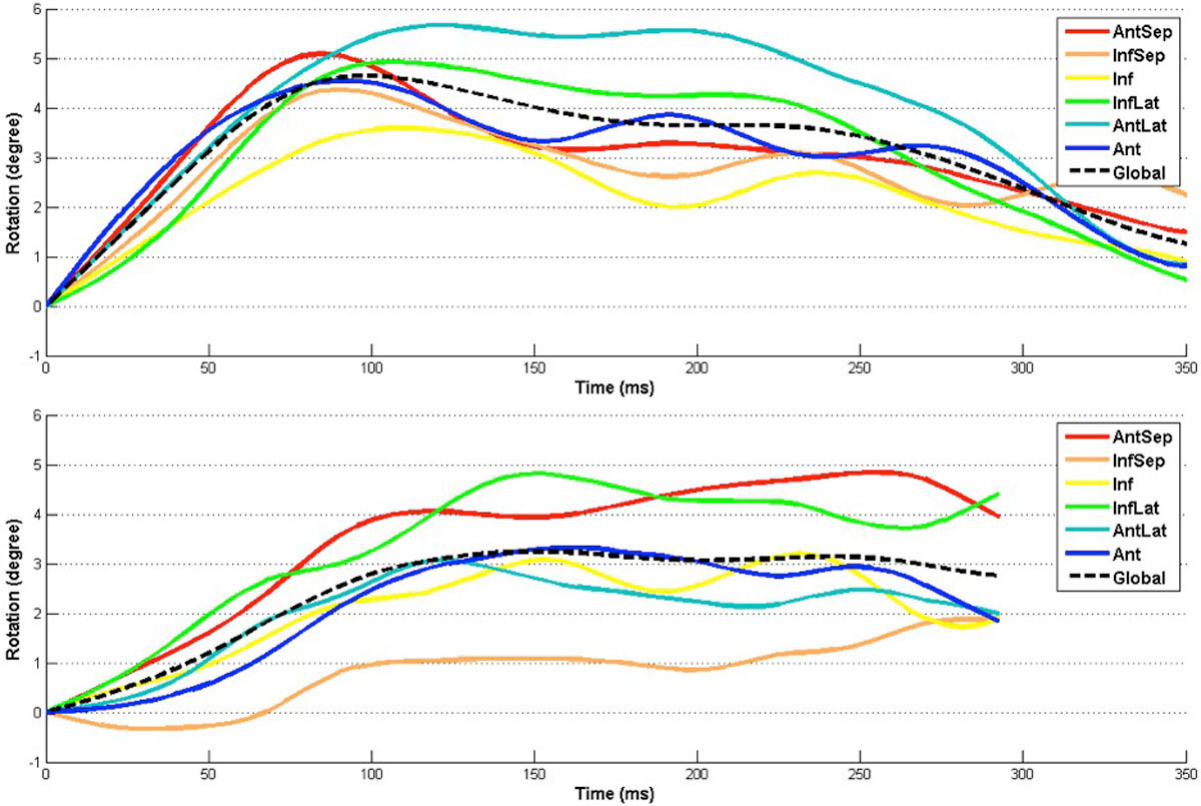
**Mean regional rotation of Mid LV in 12 healthy subject (up) and 9 cardiomyopathic patient (down)**.

The resulting mid ventricular rotation values and rotation rates in myopathic patients were decreased relative to healthy subjects in most circumferential segments (table 1 & Figure 1). Moreover, as is shown in Figure 1, the homogeneity of regional rotations at the mid level is more pronounced in healthy subjects compared with cardiomyopathic patients.

**Table 1:** Global and regional peak rotation at mid LV for healthy subjects vs.

	Mean Peak Rotation (degree)	
	**Healthy Subjects**	**CMY Patients**

InfSep	5.99 ± 4.45	3.87 ± 2.16

Inf	5.55 ± 2.59	4.79 ± 2.01

InfLat	6.41 ± 2.54	6.23 ± 2.57

AntSep	6.26 ± 4.76	6.24 ± 3.76

AntLat	6.90 ± 2.19	4.90 ± 4.44

Ant	6.07 ± 3.74	5.01 ± 4.781

Global	5.52 ± 2.75	4.37 ± 2.64

cardiomyopathic patients

## Conclusions

Initial results with high density radial tagging suggest a heterogeneous and diminished pattern of regional rotation in patients with cardiomyopathy. This may potentially reflect an imbalance in LV mechanical function and lead to decreased global rotation. Regional heterogeneity of rotation may merit further study as a myocardial functional marker. The simple and rapid calculation of regional rotation supported by radial tagging provides a unique advantage to study this parameter.

## Funding

Images were obtained through DCVI section at UCLA.

## References

[B1] van Dalen BM Kauer F Michels M Soliman OI Vletter WB van der Zwaan HB ten Cate FJ Geleijnse ML Delayed left ventricular untwisting in hypertrophic cardiomyopathy J Am Soc Echocardiogr 2009 22 1320 1326 10.1016/j.echo.2009.07.021 19815387

[B2] Saito M Okayama H Nishimura K Ogimoto A Ohtsuka T Inoue K Hiasa G Sumimoto T Funada J Shigematsu Y Higaki J Determinants of left ventricular untwisting behaviour in patients with dilated cardiomyopathy: analysis by two-dimensional speckle tracking Heart 2009 95 4 290 296 18801783 10.1136/hrt.2008.145979

[B3] van Dalen BM Soliman OI Vletter WB ten Cate FJ Geleijnse ML Left ventricular untwisting in restrictive and pseudorestrictive left ventricular filling: novel insights into diastology Echocardiography 2010 27 3 269 274 10.1111/j.1540-8175.2009.00996.x 19765059

[B4] Hansen DE Daughters GT Alderman EL Ingels NB Jr Miller DC Torsional Deformation of the Left Ventricular Mid wall in Human Hearts With Intramyocardial Markers: Regional Heterogeneity and Sensitivity to the Inotropic Effects of Abrupt Rate Changes Circ Res 1988 62 5 941 952 10.1161/01.RES.62.5.941 3282715

[B5] Buchalter MB Weiss JL Rogers WJ Zerhouni EA Weisfeldt ML Beyar R Shapiro EP Noninvasive quantification of left ventricular rotational deformation in normal humans using magnetic resonance imaging myocardial tagging Circulation 1990 81 4 1236 1244 10.1161/01.CIR.81.4.1236 2317906

[B6] Young AA Imai H Chang CN Axel L Two-dimensional left ventricular deformation during systole using magnetic resonance imaging with spatial modulation of magnetization Circulation 1994 89 2 740 752 10.1161/01.CIR.89.2.740 8313563

[B7] Rüssel IK Götte MJ Kuijer JP Marcus JT Regional assessment of left ventricular torsion by CMR tagging J Cardiovasc Magn Reson 2008 10 1 26. 10.1186/1532-429X-10-26 18505572 PMC2423368

[B8] Nasiraei-Moghaddam A Finn JP Tagging of Cardiac Magnetic Resonance Images in the Polar Coordinate System: Physical Principles and Practical Implementation Magn Reson Med 2013 Jun 26. doi: 10.1002/mrm.24839. PubMed PMID: 23804238 10.1002/mrm.24839 23804238

